# Periodontal and biochemical bone metabolism assessment on a chronic oral anticoagulation population treated with dicoumarins

**DOI:** 10.4317/medoral.21567

**Published:** 2017-02-04

**Authors:** Daniel López-Lacomba, Antonio Roa-López, Maximino González-Jaranay, Gerardo Gómez-Moreno, Gerardo Moreu

**Affiliations:** 1 Department of Haematology, Fuenlabrada Hospital, Madrid, Spain; 2 Department of Periodontology, Faculty of Dentistry, University of Granada, Spain; 3 Department of Special Care in Dentistry, Pharmacological Research in Dentistry Group, Faculty of Dentistry, University of Granada, Granada, Spain

## Abstract

**Background:**

The aim is to evaluate periodontal alteration and biochemical markers associated with bone turnover in chronic oral with dicoumarins anticoagulant treatment patients.

**Material and Methods:**

80 patients treated with oral anticoagulants were divided into 2 cohort: Group A (n=36) 6 month to 1 year with anticoagulant treatment and Group B (n=44) > 2 years with anticoagulant treatment. Clinical evaluation included: Clinical attachment level (CAL), plaque index (PI) and gingival index (GI). Analytically biochemical parameters of bone remodeling (calcium and phosphorus), formation (total acid phosphatase, alkaline phosphatase and osteocalcin) and resorption (tartrate-resistant acid phosphatase and beta-crosslaps) were evaluated.

**Results:**

High values of PI (67-100%) especially in men and in Group B were observed. Men with anticoagulation treatment length showed an increased GI (49.167 vs 78.083) while Group B women showed a decreased GI in comparison with Group A (59.389 vs 42.120). Women presented a greater average CAL than men as well as Group B vs Group A but without statistical significance. All biochemical markers were decreased respect to values of general population. Osteocalcin in GroupB women showed a statistically significant outcome vs GroupA (*p*=0.004). Acid phosphatase (total and tartrate-resistant) has a slight increase in Group B women versus Group A, and Beta-crosslap showed lower values in Group A men than Group B and slightly lower in Group A women versus Group B, without statistical significance.

**Conclusions:**

Patients showed a slight to moderate degree of periodontal affectation, especially gingivitis related to bacterial plaque. Periodontal disorders tended to be more severe in Group B. While bone remodeling showed an overall decrease with greater affectation of bone neoformation phenomena, bone destruction tended to recover and normalize in time.

**Key words:**Periodontal disease, dicoumarin, biochemical markers, bone remodeling.

## Introduction

Oral anticoagulant treatment (OAT) with dicoumarins is an increasingly common practice in general population due to their efficacy and safety (13.19/1000 inhabitants in Spanish population are treated with dicoumarins) (1). Diverse health care agents are involved in the management of oral anticoagulated patients: staff hematologist, primary attention doctor, nursing… 

Bleeding and osteoporosis are some of the adverse effects of these drugs (2). The latter has been exhaustively studied in relation to other anticoagulants such as heparins (3). However, the role of coumarins and their mechanism of interference through deactivating proteins such as GLA proteins and osteocalcin are less well known (4). The drug effect on dental treatments and on alveolar bone, and their possible repercussions for the onset or evolution of periodontitis, particularly on bone destruction, has been mentioned in several papers (5-7).

Periodontitis is one of the most frequent oral diseases. It is an inflammatory and irreversible disease, mediated by bacterial biofilm, than affects tooth-supporting tissues, inducing an alveolar bone loss, and the progressive development of clinical attachment loss. It manifests clinically as the presence of gingival inflammation, bleeding (either spontaneous bleeding or as a result of periodontal probing, tooth brushing, etc.) and the appearance of periodontal pockets. Its severity is assessed by means of the Gingival Index (GI) and Clinical Attachment Level (CAL).

Given the high prevalence of periodontal disease in the general population, its risk factors (some coinciding with the same pathologies that require treatment by oral anticoagulants), possible repercussions for the onset or evolution of other systemic alterations (diabetes, atherosclerosis,…) (8-10), and the growing strata of the population receiving anticoagulant treatment, it is important to establish the repercussions of dicoumarins for the onset, perpetuation and aggravation of periodontitis.

The objective of this study is to evaluate periodontal alteration and biochemical markers related to bone turnover in patients with chronic oral anticoagulant treatment, recording clinical periodontal parameters (plaque index -PI-, GI and CAL), biochemical markers of bone remodeling (serum ionized calcium and serum phosphorus), bone formation markers (total serum acid phosphatase, serum bone alkaline phosphatase, serum osteocalcin [Bone Gla-protein]) and bone resorption parameters (tartrate-resistant acid phosphatase and beta-crosslaps in plasma).

## Material and Methods

The study protocol was approved by the Faculty of Dentistry Ethics Committee of Granada University and followed criteria established in Helsinki Declaration for clinical trials. All participants gave their informed consent.

-Patients

Patients receiving oral anticoagulants were selected from a data base of 3220 anticoagulant patients at the Valme Area Hospital (Sevilla, Spain) who fulfilled the following inclusion criteria: patients in treatment by acenocoumarol; good patient compliance with the anticoagulant treatment, defined as least a 80% attendance at check-ups (prothrombin time - INR) (11) during a required initial period (> 6 months and < 1 year; >2 years) were into the therapeutic range established for each individual ± 10% ; whose reason for anticoagulant therapy (observe that some patients had more than one disease) did not involve risk of periodontal disease or osteoporosis ( Table 1), no other drug intake altering periodontal status or bone metabolism, and absence of risk factors for periodontal disease or osteoporosis. A total of 80 randomly selected patients were included, divided into two groups according to treatment length: Group A included patients in treatment between six months to one year; Group B patients in treatment during two years or more. Group A included 36 patients (18 men and 18 women, mean age 69.2 + 0.7) and Group B 44 patients (24 men and 20 women, mean age 68.8 + 0.8).

**Table 1:**
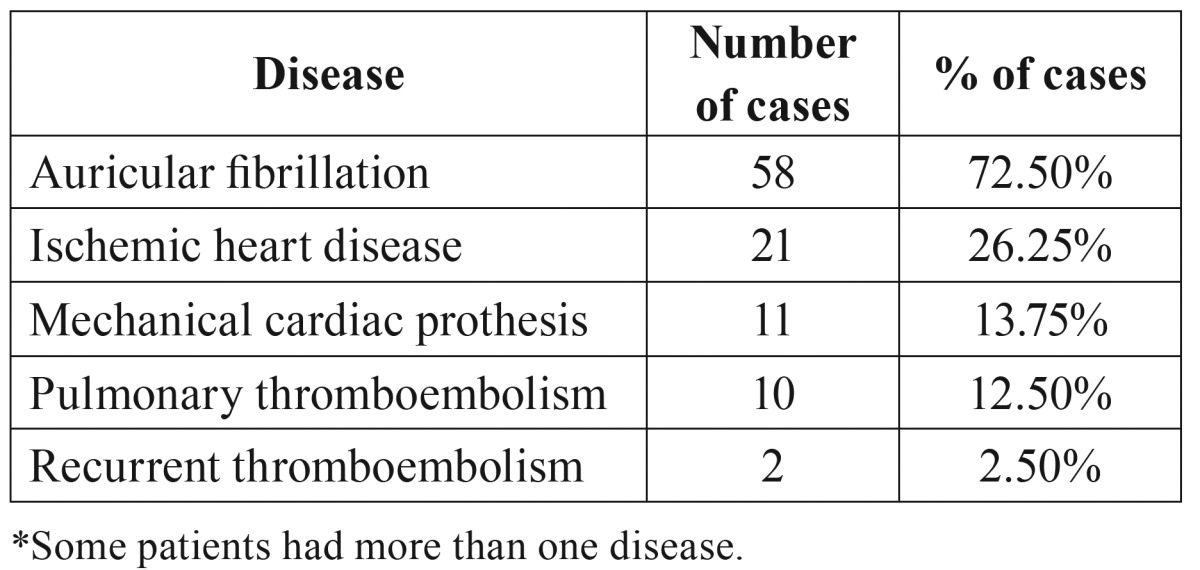
Distribution of participants according to the disease.

Given that the total population with oral anticoagulation treatment in our area is about 3200 patients it was an adequate sample size (12).

After inter-explorer calibration by means of the Cohen Kappa test, all patients underwent O’Leary’s Plaque Index (PI) (13), Ainamo and Bay Gingival Index (GI) (14), and clinical attachment level (CAL) using a periodontal probe (PCPUNC15).

- Biochemical analysis methods 

A blood sample was extracted from all subjects which underwent an anticoagulation test by means of the international normalized ratio (INR) with a STA© coagulometer and thromboplastin reagents Neoplus© (Roche Diagnostics, Basel, Switzerland). Biochemical markers of bone remodeling (serum ionized calcium and serum phosphorus), markers of bone formation (total serum acid phosphatase, serum bone alkaline phosphatase, serum osteocalcin [BGP]) and markers of bone resorption (tartrate-resistant acid phosphatase and beta-crosslaps in plasma) also were analyzed using a Hitachi Modular P800© device and Roche Diagnostic© reagents (Roche Diagnostics, Basel, Switzerland); electrophoresis on Agarose gel; immunoradiometric assay; kinetic spectrophotometry; and enzyme immunoassay. All data were perfomed by Balagué Center© laboratories (Barcelona, Spain).

- Statistical analysis

Descriptive statistics, variance analysis, and Kruskal-Wallis non-parametric test were performed using IBM SPSS Statistics 23.0 statistical package for Windows (IBM Corporation, Armonk, USA). Statistical significance was set at *p*<0.05.

## Results

- Periodontal results

Plaque index (PI): All patients showed high values between 67 and 100%. The oral hygiene was worse in men than women, especially in group B patients ( Table 2).

**Table 2:**
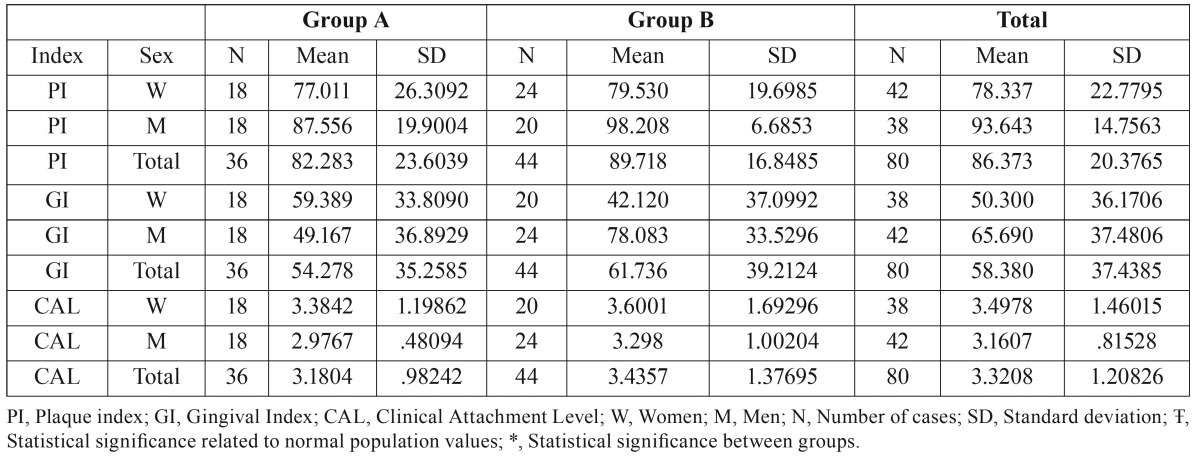
Results of periodontal variables of the patients.

Gingival Index (GI): The results showed an increase in gingival bleeding in all men patients (Groups A and B) related to the treatment anticoagulation length, while in Group B women the number of bleeding sites decreased in comparison with the others groups ( Table 2).

Clinical Attachment Level (CAL): Mean CAL was greater in women than men, and both men and women showed slightly greater CAL in Group B than Group A; neither of them were statistically significant different ( Table 2).

-Biochemical results

Calcium and phosphorus values were similar in the two groups although mean levels were lower compared with normal healthy population ( Table 3).

**Table 3:**
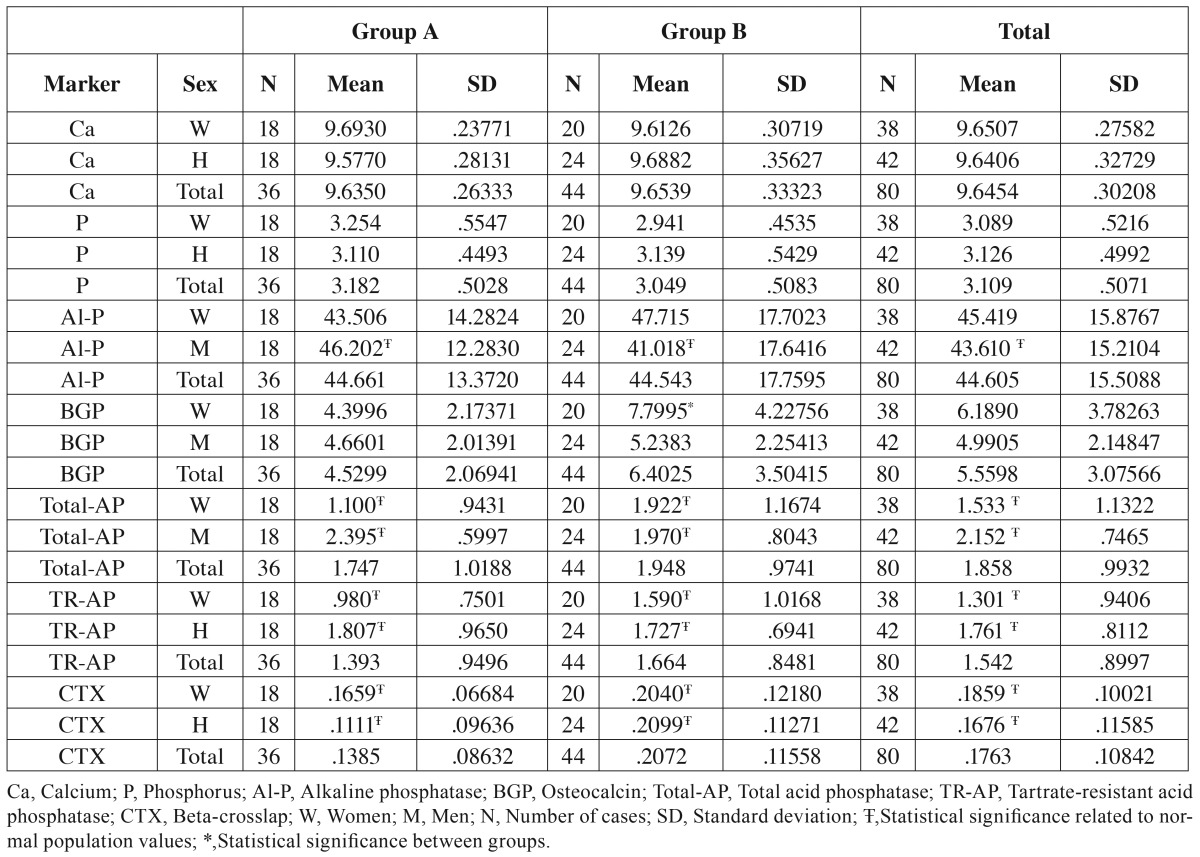
Results of biochemical determinations of patients.

Total alkaline phosphatase: In women no statistically significant differences were identified (comparing Groups A and B or both groups with the estimated values for general population). However, in the case of male patients, values were significant statistically lower than general population in both groups, although higher in Group B than Group A ( Table 3).

Osteocalcin: Both groups and both genres presented lower values than general population. Although men showed similar values in both groups, women showed a statistically significant improvement in Group B (*p*=0.004) vs. Group A ( Table 3).

Acid phosphatase values, both total acid phosphatase and tartrate-resistant acid phosphatase, showed statistically significant reductions in all groups. In men there was no relation of anticoagulant treatment length. There were lower values in Group A women, however these values increased in Group B ( Table 3).

Beta Crosslap: In all cases there was a statistically significant decrease compared with normal levels in general population. Values in men were lower in Group A than Group B. In women there were no statistically significant differences between them according to anticoagulation treatment length, although values were slightly lower in Group A ( Table 3).

## Discussion

As in previous researches, this study chose to evaluate serum biochemical markers and bone remodeling parameters (calcium, phosphorus, alkaline phosphatase, beta-crosslaps, total acid phosphatase and tartrate-resistant acid phosphatase) (15). These methods (16) are safe, reliable and more feasible than invasive procedures – as bone biopsies are - only requiring a blood or urine sample so given the ease of taking samples, continuous evaluations of bone metabolism can be conducted easily.

Results for all these parameters obtained from patients taking the anticoagulant warfarin suggest that bone remodeling is generally reduced, with increased affectation of bone neoformation, while bone destruction tends to recover and return to a physiological steady state. Previous studies have evaluated vitamin K and bone mineral density in patients administered long term warfarin following cerebral ischemic attack, finding that mineral bone density is significantly lower between long-term warfarin patients than non-treated patients (2,17).

The biochemical action’s mechanism of warfarina and coumarins are similar and results from its anti vitamin K effect. Their principal difference is settled in their different biodisponibility and half life in the organism, more long and stable for the warfarin. In Spain the predominant drugs are coumarins (Sintrom®) and in practice the use of warfarin is restricted to patients with metallic prosthetic valves, a minority population group, and only when his control with coumarins is very irregular (18,19).

 It is important to remember that biochemical data and clinical disorders are not simultaneous, so that it may take a long evolution time for an increase in biochemical status to translate into clinical pathology. This action is also encountered in our study.

We select two differents cohorts both with enough time for develop changes in bone metabolism. The first (Group A) has a minimum of six months for that. The length of anticoagulant treatment in some pathologies (like lung thromboembolism) has usually between 6-12 months so that we set up the limit of the first group in one year. For avoid overlap and can evaluate changes after a significantly longer period of treatment the second group received it for at least 2 years (Group B). Most of patients with cardiac diseases meet this condition.

From the biochemical point of view, the most important statistically significant data in our study were those indicating the ongoing reduction in carboxylated osteocalcin. It means that the main component of the osteoid matrix is reduced and so it can be inferred that bone remodeling phenomena with new bone formation was probably reduced or delayed. Other parameters usually affected in these cases – calcium, phosphorous, and total alkaline, and bone phosphatase – which are linked to osteocalcin, were also found to be displaced although they did not decrease to values that could be considered pathological or significant from the clinical point of view.

Bone resorption parameters – tartrate-resistant acid phosphatase, and beta-crosslaps – presented lower average values than would be considered normal between the general population, a finding that is coherents with the known effect of osteocalcin on osteoclast activity and differentiation.

PI data indicated poor plaque control in all cases, showing values of over 67%, with worse oral hygiene between men than women. This genres difference agrees with the research into populations in the developed world (20). Treated patients, for whom bleeding is more frequent as a result of anticoagulation treatment, may have more fear of the possibility of bleeding during tooth brushing, which in turn could lead them to avoid brushing leading to decreased removal of bacterial plaque, hence the higher plaque index seen in Group B than Group A (21), like patients reported during periodontal evaluation.

As for gingival bleeding, the results showed an increase rate between male patients in relation to the length in anticoagulant treatment, which could be related to the increased PI and poorer oral hygiene outlined above (22). But between the female population, as anticoagulant treatment time increased (Group B) GI values decreased. Although this might appear paradoxical, it could be the result of a relation with the osteocalcin and/or acid phosphatase levels found in the study. Although other authors such as Bullón related increased serum osteocalcin with deterioration of periodontal status and lower treatment response regarding clinical attachment level (CAL) (23,24), the present study population showed lower values for biochemical parameters in all patients but slight increases in serum levels would bring them close to minimum normal values therefore producing an improvement in gingival bleeding data.

With regard to clinical attachment level, no significant differences were found between men and women. All cases showed moderate increases in CAL (no greater than 3.5 mm), with data that agree with epidemiological studies in socioculturally similar populations and with epidemiological studies conducted in Spain, although these were measured according to the community periodontal index of treatment needs (CPITN) (25-27). Comparing men and women, it was seen that for both genres probing depth was slightly greater between Group B patients than Group A, although differences were not statistically significant. This could be related to deterioration over time, independently of the demineralization biochemical markers and connective tissue destruction involved in the evolution process of periodontal pocket depth (28).

## Conclusions

The study population showed a slight to moderate degree of periodontal affectation, the most common disorder being gingivitis related to bacterial plaque. Periodontal disorders tended to be more severe in patients who had been taking anticoagulants for longer.

Biochemical results suggest that bone remodeling may have an overall decrease with greater affectation of bone neoformation phenomena but that bone destruction tends to recover and normalize with time.

As anticoagulant patients suffer chronic conditions and so are subject to regular check-ups, we think that there are strong reasons for oral hygiene instruction to be included in health care services for oral anticoagulation patients. This would avoid increasing periodontal lesion or complications that require subsequent invasive treatments that could affect anticoagulant management and in turn have repercussions for systemic pathologies.
